# Clinical risk predictors for differentiated thyroid cancer management: what is new?

**DOI:** 10.20945/2359-3997000000110

**Published:** 2019-02-01

**Affiliations:** 1 Universidade Federal do Rio de Janeiro Universidade Federal do Rio de Janeiro Departamento de Endocrinologia Brasil Departamento de Endocrinologia, Universidade Federal do Rio de Janeiro (UFRJ). Faculdade de Medicina, Universidade Federal do Estado do Rio de Janeiro (Unirio), Rio de Janeiro, RJ, Brasil; Universidade Federal do Estado do Rio de Janeiro Universidade Federal do Estado do Rio de Janeiro Faculdade de Medicina Rio de Janeiro RJ Brasil

Management of differentiated thyroid cancer (DTC) is highly dependent on risk stratification (1,2). An initial assessment of the risk of mortality and the risk of persistent/recurrent disease are required to guide treatment, follow-up and to set appropriate patients and physicians expectations with regard to the clinical outcomes after initial therapy. Therefore, much effort has been made for the improvement of long-term clinical risk predictors based on the information available at the time of diagnosis. In this issue of the *Archives of Endocrinology and Metabolism*, two very interesting papers were published concerning this subject.

Nava and cols. (3) evaluated the impact of the updated American Joint Committee on Cancer; Tumor, Lymph Nodes, Metastasis (AJCC/TNM) system staging criteria on the prediction of persistent disease in DTC. The major changes from the updated TNM 8^th^ edition were: the age cutoff used for staging was increased from 45 to 55 years of age at diagnosis; minor extrathyroidal extension detected only on histological examination was removed from the definition of T3 disease and therefore has no impact on either T category or overall stage; T3a is a new category for tumors > 4 cm confined to the thyroid gland; T3b is a new category for tumors of any size demonstrating gross extrathyroidal extension into strap muscles (sternohyoid, sternothyroid, thyrohyoid, or omohyoid muscles); level VII lymph nodes, previously classified as lateral neck lymph nodes (N1b), were reclassified as central neck lymph nodes (N1a); and the presence of distant metastases in older patients is classified as stage IVB disease rather than stage IVC disease (4).

In the study of Nava and cols., the application of TNM 8^th^ edition resulted in 37% (155/419) of DTC patient's down staged and improved the prediction of poor outcomes compared to the TNM 7^th^ edition. The re-classification for lower risk categories (stage I or stage II disease) observed in this cohort of Brazilian patients with DTC is the expected effect of the updated 8^th^ TNM edition and allowed the appropriate classification of the majority of thyroid cancer patients as being at low risk recurrent/persistent structural disease during the follow-up. On the other hand, patients who remained classified as stage IV and III based on the 8^th^ edition had more advanced disease and presented with higher rates of incomplete response to therapy. The TNM 8^th^ edition had a better performance in predicting disease outcomes based on dynamic risk stratification using the response to therapy (1) than the 7^th^ edition. Interestingly, even though the AJCC/TNM was mainly designed to predict initial estimates of disease specific mortality (1), the present study demonstrated that the 8^th^ edition provided a satisfactory initial estimates risk of recurrent/persistent disease (3).

The impact of the updated TNM staging system was more notable for older patients. The modification in the age cutoff value was based in an international multi-institutional validation study of 9484 patients, which included patients from Brazil (cohort from Rio de Janeiro) and demonstrated that an increase in the age cutoff from 45 to 55 years of age at diagnosis down staged 12% of patients and was associated with a 10-year disease-specific survival of 98% in the down staged group (5). Similarly, the change in the age at diagnosis cutoff from 45 to 55 years was responsible for the re-classifications of 20.8% (87/419) of patients in the cohort of Nava's study (3). Disease-specific survival could not be evaluated due to the low mortality rate observed during follow-up. Taken together, these findings endorse the new 8^th^ edition of the AJCC/TNM staging system as a better clinical risk predictor for the management of DTC.

Management of DTC would be also improved with reliable clinical risk predictors available before initial surgical procedure. For this purpose, the study published in this issue by Lima and cols. (6) evaluates the Bethesda system for reporting thyroid cytopathology (7) as a predictor of aggressive features in DTC. The Bethesda system was originally designed to predict the risk of malignancy of thyroid nodules based on the cytopathological findings and to provide clinical management recommendations for each of its six diagnostic categories. The Bethesda System was revised in 2017 inspired by new data available since it was first described 2009, the use of molecular testing as an adjunct to cytopathologic examination and the reclassification of the noninvasive follicular variant of papillary thyroid carcinoma as noninvasive follicular thyroid neoplasm with papillary-like nuclear features (NIFTP) (7).

The study by Lima and cols. (6) evaluated 153 patients with DTC and assessed the relationship of their preoperative fine needle aspiration diagnosis using the Bethesda system with the presence of aggressive features in histopathology, risk stratification staging systems and clinical outcomes. They found a positive relationship of Bethesda V (suspicious for malignancy) and VI (malignant) with vascular invasion and lymph node metastasis, which are associated with increased risk of recurrent/persistent disease in DTC. They also described a positive relationship of Bethesda V and VI with extrathyroidal extension, but the real impact of this finding relates do the extent of the tumor invasion to extrathyroidal structures and this was not described in the study. There was no statistically significant association between the Bethesda system categories and the American Thyroid Association (ATA) risk stratification (2) or TNM staging system considering the entire cohort. Nevertheless, most of ATA high-risk patients (54.5%) had a Bethesda V or VI result. These data suggest that the preoperative cytopathological diagnosis of Bethesda V and VI could imply not only in higher risk of malignancy (7), but also in an increased risk of DTC with aggressive features.

The preoperative diagnosis of Bethesda II was observed in a higher than expected number of patients with DTC (29.4%). Fortunately, the vast majority of DTC patients that presented with Bethesda II evolved with excellent response to therapy and no evidence of disease (92.1%) (6). According to the Bethesda system the implied risk of malignancy for the diagnostic category II (benign) is of 0-3% and the recommended clinical approach is clinical and sonographic follow-up (7). The results of the present study suggest a very good prognosis for DTC with a Bethesda II result, what could minimize the clinical implication of the false negative result for malignancy of the cytopathology.

The study of Lima and cols. (6) provides a new insight for the clinical utility of the Bethesda System, in which the preoperative cytopathology diagnosis would indicate the estimated risk of malignancy in thyroid nodules and predict the risk of aggressive features in DTC. Nevertheless, this is a small pilot study, with a retrospective design, and future studies are required to confirm these findings.

The new evidence provided by these studies indicates that the long-term clinical risk prediction of DTC could be improved, based on refined findings of the preoperative cytopatholgy, histopathological data and risk stratification systems. Additionally, molecular biology will also have an important role. More studies are necessary in this field. The advances in the risk-adapted management of DTC would allow a more accurate individualized approach and better tailor treatment and follow-up strategies for patients with DTC.
